# Guidelines for Reproductive Medicine in Japan

**DOI:** 10.1002/rmb2.12483

**Published:** 2022-08-24

**Authors:** Mitsutoshi Yamada, Tomonori Ishikawa, Takeshi Iwasa, Hajime Oishi, Satoko Osuka, Kenji Oka, Shuichi Ono, Masanori Ono, Makoto Orisaka, Haruhiko Kanasaki, Yasushi Kawano, Kazuhiro Kawamura, Hiroshi Kishi, Fuminori Kimura, Shinnosuke Kuroda, Akira Kuwahara, Hideyuki Kobayashi, Akira Komiya, Hidekazu Saito, Kenji Sato, Suguru Sato, Koji Shiraishi, Hiromitsu Shirasawa, Tatsuya Suzuki, Yasushi Takai, Seido Takae, Toshifumi Takahashi, Tsuyoshi Takiuchi, Masahito Tachibana, Isao Tamura, Hiroshi Tamura, Seung Chik Jwa, Tsuyoshi Baba, Miyuki Harada, Tetsuya Hirata, Atsushi Fukui, Yusuke Fukuda, Shinichiro Fukuhara, Tetsuo Maruyama, Yasushi Yumura, Osamu Yoshino, Yasushi Hirota, Akira Tsujimura, Naoaki Kuji, Yutaka Osuga

**Affiliations:** ^1^ Department of Obstetrics and Gynecology Keio University School of Medicine Shinjuku‐ku Tokyo Japan; ^2^ Tokyo Medical & Dental University Graduate School of Medical and Dental Sciences Bunkyo‐ku Tokyo Japan; ^3^ Institute of Biomedical Sciences Tokushima University Graduate School Tokushima Japan; ^4^ National Center for Global Health and Medicine Shinjuku‐ku Tokyo Japan; ^5^ Nagoya University Hospital Nagoya‐shi Aichi Japan; ^6^ Mirai ART Oka Ladies Clinic Nagano‐shi Nagano Japan; ^7^ Shinjuku ART Clinic Shinjuku‐ku Tokyo Japan; ^8^ Tokyo Medical University Shinjuku‐ku Tokyo Japan; ^9^ University of Fukui Yoshida‐Gun Fukui Japan; ^10^ Shimane University Hospital Izumo‐shi Shimane Japan; ^11^ Oita University Faculty of Medicine Yufu‐shi Oita Japan; ^12^ Department of Obstetrics and Gynecology International University Health and Welfare School of Medicine Naritashi Chiba Japan; ^13^ The Jikei University School of Medicine Minato‐ku Tokyo Japan; ^14^ Department of Obstetrics and Gynecology Shiga University of Medical Science Otsu Shiga Japan; ^15^ Department of Urology, Reproduction Center Yokohama City University Medical Center Yokohama‐shi Kanagawa Japan; ^16^ Ladies Clinic Cosmos Kochi‐shi Kochi Japan; ^17^ Toho University, School of Medicine Ota‐ku Tokyo Japan; ^18^ Chiba University Graduate School of Medicine Chiba‐shi Chiba Japan; ^19^ Umegaoka Women's Clinic Setagaya‐ku Tokyo Japan; ^20^ Tokai University Hospital Isehara Kanagawa Japan; ^21^ Ogikubo Hospital Niji Clinic Suginami‐ku Tokyo Japan; ^22^ Yamaguchi University Graduate School of Medicine Ube‐shi Yamaguchi Japan; ^23^ Akita University Hospital Akita‐shi Akita Japan; ^24^ Jichi Medical University Shimotsuke‐shi Tochigi Japan; ^25^ Saitama Medical Center Saitama Medical University Kawagoe‐shi Saitama Japan; ^26^ Department of Obstetrics and Gynecology St. Marianna University School of Medicine Kawasaki‐shi Kanagawa Japan; ^27^ Fukushima Medical University Fukushima‐shi Fukushima Japan; ^28^ Osaka University Graduate School of Medicine Suita Japan; ^29^ Department of Obstetrics and Gynecology Tohoku University Hospital Sendai Miyagi Japan; ^30^ Department of Obstetrics and Gynecology Saitama Medical University Iruma‐gun Saitama Japan; ^31^ Sapporo Medical University Sapporo‐shi Hokkaido Japan; ^32^ The University of Tokyo Bunkyo Tokyo Japan; ^33^ Doai Kinen Hospital Sumida‐ku Tokyo Japan; ^34^ Hyogo Medical University Nishinomiya‐shi Hyogo Japan; ^35^ Toho University Omori Medical Center Ota‐ku Tokyo Japan; ^36^ University of Yamanashi Chuo Yamanashi Japan; ^37^ Juntendo University Urayasu Hospital Urayasu‐shi Chiba Japan; ^38^ Faculty of Medicine Juntendo University Bunkyo‐ku Tokyo Japan; ^39^ Nara Medical University Kashihara Nara Japan; ^40^ Kameda Medical Center Kamogawa‐shi Chiba Japan; ^41^ St. Luke's International Hospital Chuo‐ku Tokyo Japan

**Keywords:** assisted reproductive technology, clinical question, guideline, infertility, recommendation

## 1 INTRODUCTION

Assisted reproductive technology (ART) is a relatively new field compared with other medical treatments; so far, the evidence that forms the basis of medical treatment and treatment guidelines has yet to be sufficiently established. In addition, there has been a tendency in the past for new techniques to be introduced into on‐the‐job medical care without evidence being established. As we entered the 21st century, evidence gradually increased, and the need for accurate evaluations of medical technologies has increased worldwide, resulting in the creation of multiple guidelines in Western countries in the last 10 years or so.

Based on the “Law concerning special provisions of the Civil Code concerning the provision of assisted reproductive technology and the parent–child relationship of children born as a result” promulgated on December 11, 2020, in Japan, ART has been covered by insurance from April 2022 as a concrete measure (Act No. 76 of 2020). As a reference when considering insurance coverage, the government has requested the Japan Society for Obstetrics and Gynecology, which deals with obstetrics and gynecology in general, and the Japan Society for Reproductive Medicine, which deals with ART, including male infertility, to create medical treatment and treatment guidelines. Within this context, it was decided that medical care and treatment guidelines be created based on the actual medical conditions and evidence in Japan.

The authors of the Guidelines Development Committee have contributed significantly to the drafting of the guidelines. A draft was published on the website of the Japan Society for Reproductive Medicine, and opinions were solicited from members, resulting in repeated amendments. Following deliberation by the evaluation committee, the content of the amendments was discussed, and on May 26, 2021, the original draft first proof composition policy was confirmed. In November 2021, the first edition of “Assisted Reproductive Technology Guidelines 2021,” consisting of 40 Clinical Questions (CQ), and answers was published.

Since this guideline is written in Japanese, there were some inconveniences for those who are not native speakers of the Japanese language. An English version was created to overcome this problem. This book includes “commentary,” “actual conditions,” “usage of drugs,” “reference list,” and “charts,” but these parts have been omitted owing to space limitations. Furthermore, the description of the general infertility area has been excluded from this document to avoid duplication with the Obstetrics and Gynecology Practice Guidelines Gynecology Outpatient Edition 2020, which was edited and supervised by the Japan Society of Obstetrics and Gynecology and the Japan Association of Obstetricians and Gynecologists.

As the Japan Society for Reproductive Medicine participated in the editing of these guidelines, we would like to add that we take great responsibility as an organization of medical researchers/medical professionals in helping patients suffering from infertility and hope that we can contribute to the healthy birth and growth of children who will usher our country into the future.

## 2 IMPLICATIONS OF “A,” “B,” AND “C” RECOMMENDATION LEVELS

Recommendation levels (strength) “A,” “B,” and “C” are shown in each CQ and answer. These recommendation levels were created by comprehensively considering the clinical usefulness, evidence, penetrance, and medical economic viewpoint of the recommended testing and treatment methods. Therefore, the answers are not necessarily based on “evidence.” When evidence is lacking, a decision is reached by considering the balance between benefit and harm, values and preferences, penetrance, costs, and resource utilization. The recommendation levels are interpreted as follows:

A: Highly recommended (for implementation, etc.),

B: Recommended (for implementation, etc.),

C: Considered (for implementation, etc.).

Factors that determine the recommendation level include the quality of evidence, balance between benefits and harm, values and preferences, penetrance, cost, and resource utilization. The recommendation level is likely to be higher as the overall evidence increases in strength, if the desired effects outweigh the undesired effects and the gap between the two increases, and when the costs resources correspond to the net benefits.

### 2.1 Terminology

Ovulation induction is defined as pharmacological treatment with the intention of inducing normal ovulatory cycles of women with anovulation or oligo‐ovulation for timed intercourse or intrauterine insemination (non‐ART). Ovarian stimulation is defined as pharmacological treatment with the intention of inducing the development of ovarian follicles for IVF/ICSI (ART).

### 2.2 Contents

Chapter A. Desirable equipment and personnel for Assisted Reproductive Technology (ART) (CQ001, CQ002).

Chapter B. Indications for ART (CQ003–CQ005).

Chapter C. Ovarian stimulation in ART (CQ006–CQ014).

Chapter D. Prevention and treatment of ovarian hyperstimulation syndrome (OHSS) (CQ015).

Chapter E. Embryo culture and manipulation (CQ016–CQ023).

Chapter F. Fresh embryo transfer and frozen embryo transfer (CQ024–CQ028).

Chapter G. Add‐on medical treatment (CQ029–CQ035).

Chapter H. Psychological support and counseling (CQ036).

Chapter I. Male infertility (CQ037–040).

## 3 CLINICAL QUESTIONS AND ANSWERS

### 3.1 **Chapter A**. Desirable equipment and personnel for Assisted Reproductive Technology (ART).

**CQ001**: What are the conditions inside the egg collection/culture room?

Answers

1. Install high‐efficiency particulate air (HEPA) filters and control volatile organic compounds to optimize the air quality of the culture room. (A)
2. The culture room shall have machines and locking equipment related to egg/sperm/embryo processing/culture and cryopreservation. (A)
3. Install HEPA filters and control volatile organic compounds to optimize air quality of the operation room for oocyte retrieval. (C)
4. The operation room for oocyte retrieval should be equipped with equipment related to the oocyte retrieval procedure: operating table, ultrasonic tomography device, oxygen inhaler, aspirator, biological monitor, and emergency resuscitation set. (A)
5. The oocyte retrieval/culture room should contain manuals for medical treatments/procedures and equipment operation. (A)
6. Medical treatment/procedure and equipment operation inspections should be recorded in the oocyte retrieval/culture room. (A)
7. The medical treatment/procedure in the oocyte retrieval/culture room or its environment should be safely managed. (A)
8. An emergency backup plan in the culture room should be developed. (A)

**CQ002**: What are the qualifications of the attending physician? What are the staffing requirements for non‐physician personnel?

Answers

1. Attending physicians must meet the following conditions: (A)

- Be a specialist in obstetrics and gynecology certified by the Japan Society of Obstetrics and Gynecology and has been engaged in infertility treatment for more than two years after becoming a specialist.
- Have worked at a registration facility for in vitro fertilization/embryo transfer (registered facility for ART) of the Japan Society of Obstetrics and Gynecology for one year or more or have received training for one year or more and have learned the techniques of in vitro fertilization/embryo transfer.
- Be full‐time physicians.
- Ideally, be reproductive medicine specialists certified by the Japan Society for Reproductive Medicine.

2 Non‐physician personnel must meet the following conditions. (A)

- One or more nurses.
- One or more technicians (physician or embryo culture specialists) who are able to handle embryos. Ideally, a facility that collects eggs for 150 cycles or more per year should have two or more embryo culture specialists.

### 3.2 Chapter B. Indications for ART

**CQ003**: Is in vitro fertilization (IVF)/intracytoplasmic sperm injection effective for achieving pregnancy? What are the optimal number of trials and eligibility criteria for IVF/intracytoplasmic sperm injection?

Answers

1. IVF is effective as a treatment for unexplained infertility. (A)
2. In IVF, the live birth rate per treatment decreases as the female increases in age. (A)
3. In IVF, the cumulative live birth increases as the number of treatments increases, and is affected by female age, cause of infertility, and treatment. (B)
4. IVF must be performed in accordance with the opinions and announcements of the Japan Society of Obstetrics and Gynecology. (A)

**CQ004**: In what cases would it be allowed for one to directly proceed to IVF/intracytoplasmic sperm injection? What tests and treatments are needed in each case?

Answers

1. IVF is performed in cases of infertility wherein both fallopian tubes have lost their functions. (A)
2. For severe male infertility cases, a urological examination is performed. (A)
3. In the case of severe male infertility, IVF/intracytoplasmic sperm injection is performed. (B)
4. When surgically collected sperm from the testis or epididymis is used for treatment, intracytoplasmic sperm injection is performed. (A)
5. In the initial treatment when ART is performed in cases other than male infertility cases—such as unexplained infertility—so‐called split insemination may be considered. (B)

**CQ005**: What are the indications for testicular sperm extraction (TESE)? Is TESE with intracytoplasmic sperm injection effective?

Answers

1. Microdissection TESE (micro‐TESE) is recommended as a sperm retrieval technique for intracytoplasmic sperm injection in patients with non‐obstructive azoospermia. (A)
2. Micro‐TESE is also presented as an optional technique for fertility preservation in non‐obstructive azoospermia in cancer patients treated with testicular toxic drugs. (A)

### 3.3 Chapter C. Ovarian stimulation in ART

**CQ006**: Is the evaluation of the ovarian reserve effective in selecting the gonadotropin dose for ovarian stimulation?

Answer

1. Antral follicle count (AFC) and anti‐Müllerian hormone (AMH) measurements are useful for predicting ovarian response during ovarian stimulation. (A)

**CQ007**: Is hormone pretreatment effective for ART?

Answer

1. Estrogen, progestin, and estrogen/progestin combination drug in the pretreatment cycle are used for the purpose of adjusting the treatment cycle; however, in each case, the benefits and disadvantages incurred by the patient are carefully taken into consideration. (A)

**CQ008**: Are gonadotropin‐releasing hormone (GnRH) antagonists more effective than GnRH agonists in ovarian stimulation for high responders?

Answers

1. When controlled ovarian hyperstimulation with gonadotropin is used in a high responder, reducing the dose of gonadotropin is effective in decreasing the risk of ovarian hyperstimulation syndrome (OHSS). (A)
2. The GnRH antagonist cycle can decrease the risk of OHSS at a similar pregnancy rate as the GnRH agonist cycle. In particular, in polycystic ovary syndrome (PCOS), controlled ovarian hyperstimulation with a GnRH antagonist is performed instead of the GnRH agonist cycle to prevent OHSS. (B)

**CQ009**: Is adding a blood test to the ultrasound tomography effective in monitoring follicle development during the IVF cycle?

Answers

1. Follicle development should be appropriately monitored by ultrasonography. (A)
2. If monitoring through ultrasonography is difficult or if excessive or poor ovarian response to ovarian stimulation is suspected, use a blood test in addition to the ultrasonography. (B)

**CQ010**: What are the precautions for ovarian stimulation by (ovarian stimulation protocol/LH surge suppression method/test) IVF? Is follicle‐stimulating hormone (FSH) more effective for ovarian stimulation than human menopausal gonadotropin (hMG)?

Answer

1. There is no clear difference in efficacy or safety between FSH and hMG. (A)

**CQ011**: Is natural cycle/mild ovarian stimulation effective for ovarian stimulation?

Answers

1. There is no difference in live birth rates between natural cycle IVF (nIVF) or modified natural cycle IVF (mnIVF) and IVF based on controlled ovarian stimulation. (C)
2. The use of nonsteroidal anti‐inflammatory drugs for ovulation inhibition in mnIVF cycle and in mild ovarian stimulation (mild IVF) cycle is based on the administration of moderate doses of FSH and GnRH antagonists can be recommended. (B)
3. Mild IVF in normal responders is as effective as IVF based on controlled ovarian stimulation. (A)
4. There is not enough evidence to recommend mild IVF for normal or high responders, either with clomiphene citrate (CC) alone or with a moderate dose of gonadotropin. (C)
5. For low responders, performing mild IVF based on CC alone or in combination with CC and moderate doses of gonadotropin is comparable in efficacy to IVF based on controlled ovarian stimulation. (A)

**CQ012**: Is letrozole (LTZ) effective for ovarian stimulation in PCOS? Is metformin (Met) effective for ovarian stimulation in PCOS? Is LTZ effective for ovarian stimulation in unexplained infertility? Is gonadotropin therapy with LTZ more effective for ovarian stimulation than gonadotropin therapy with clomiphene citrate (CC)?

Answers

1. For ovulation induction, LTZ is effective in non‐ART treatment for PCOS. (A)
2. Met is effective for ovulation induction in non‐ART and for ovarian stimulation in ART treatment for some cases of PCOS. (B)
3. For ovarian stimulation, LTZ is effective in non‐ART treatment for unexplained infertility. (B)
4. Gonadotropin therapy combined with LTZ is effective for ovarian stimulation in infertile patients with estrogen‐sensitive malignancies. (A)
5. For ovarian stimulation in ART for infertility, gonadotropin therapy with LTZ is as effective as gonadotropin therapy with CC. (B)

**CQ013**: Is progestin‐primed ovarian stimulation (PPOS) effective for ovarian stimulation in patients with unexplained infertility?

Answers

1. PPOS is an equally effective ovarian stimulation protocol in terms of the number of eggs collected, clinical pregnancy rate, and live birth rate premised on freeze–thaw embryo transfer, as compared to the GnRH agonist and GnRH antagonist protocols. (A)
2. PPOS has a significantly lower risk of developing OHSS than the GnRH agonist or antagonist method. (A)
3. There were no significant differences in birth defects, low birth weight, or preterm birth rate between children born with PPOS and those born with the GnRH agonist method. (B)

**CQ014**: Are GnRH agonists more effective than human chorionic gonadotropin (hCG) for egg maturation and OHSS avoidance?

Answers

1. In the GnRH antagonist cycle, a GnRH agonist trigger is more effective in preventing the onset and aggravation of OHSS than a hCG trigger. (A)
2. Only hCG can be used as a trigger in the GnRH agonist cycle. (A)
3. The method of using both hCG and GnRH agonist as a trigger can be applied when the fertilization rate is low or there is a history of a large number of immature eggs. (C)

### 3.4 Chapter D. Prevention and treatment of ovarian hyperstimulation syndrome (OHSS)

**CQ015**: Is interventional treatment effective in preventing the onset and aggravation of OHSS associated with ART?

Answers

1. It is important to recognize the risk factors to prevent the onset. (A)
2. To prevent the onset and aggravation of OHSS associated with ART, controlled ovarian stimulation by the coasting method is considered, especially for patients at risk of OHSS. (B)
3. For patients at risk of OHSS, prophylaxis with various drugs is considered. (B)
4. Freeze‐all strategy is also considered for patients at risk of OHSS. (A)

### 3.5 Chapter E. Embryo culture/embryo manipulation

**CQ016**: Is embryo culture effective to establish pregnancy?

Answers

1. Embryo culture is effective in establishing pregnancy. (A)
2. The effectiveness of sequential media and single media used for embryo culture is the same. (B)
3. There is no evidence that a particular culture is superior. (B)
4. In addition to chemical factors, such as culture medium and oxygen partial pressure, the optimal culture environment for embryos involves physical factors, such as temperature, pH, and embryo manipulation. (B)
5. In ART, both the cleavage and blastocyst stages have advantages and disadvantages. (B)

**CQ017**: What are the indications and effects of in vitro maturation (IVM)?

Answers

1. In ART for PCOS patients, controlled ovarian stimulation is performed while taking preventive measures against the onset of OHSS. (A)
2. IVM is acceptable if the risk of developing OHSS is particularly high. (B)

**CQ018**: Is a time‐lapse incubator effective in assessing embryonic development? Is time‐lapse incubation effective in improving IVF performance?

Answers

1. A lot of morphological embryo information can be obtained by continuously monitoring embryo development. (B)
2. Both the improvement of the embryo culture environment by the time‐lapse incubator and the selection of high‐quality embryos based on a large amount of morphological information can improve the pregnancy rate and live birth rate by IVF. (C)

**CQ019**: What is the indication and effectiveness of preimplantation genetic testing for aneuploidy (PGT‐A)?

Answers

1. There is no clear evidence that ART with PGT‐A improves the cumulative pregnancy and live birth rates compared with those of ART without PGT‐A. (B)
2. ART with PGT‐A is useful for the purpose of avoiding miscarriage with recurrent implantation failure. (B)

**CQ020**: Is elective single embryo transfer (SET) useful for reducing the risk of multiple pregnancies?

Answers

1. SET is useful for reducing the risk of multiple pregnancies. (A)
2. As a general rule, embryo transfer is SET. (A)
3. Dual embryo transfer (DET) is allowed for women over the age of 35, or women who have failed to conceive more than once in a row. (B)

**CQ021**: Is assisted hatching effective for ART?

Answers

1. There are several, albeit small, studies showing that the clinical pregnancy rate was improved by assisted hatching. Currently, the application of assisted hatching is permitted to be driven on the basis of the case. (B)
2. It cannot be ruled out that assisted hatching may increase the chance of multiple pregnancies. (C)

**CQ022**: Is sperm selection (e.g., intracytoplasmic morphologically selected sperm injection (IMSI) and physiological intracytoplasmic sperm injection (PICSI)) effective for ART with a high‐magnification microscope?

Answer

1. Performing advanced sperm selection techniques in ART may be considered, but there is insufficient evidence to determine the positive effect of IMSI or PICSI on ART. (C)

**CQ023**: Is artificial oocyte activation (AOA) effective for ART?

Answers

1. AOA using Ca ionophore is an effective treatment for fertilization failure after intracytoplasmic sperm injection (ICSI). (B)
2. There are no significant differences in birth defects, obstetric prognosis, or neonatal prognosis between ICSI and ICSI‐AOA. (B)

### 3.6 Chapter F. Fresh embryo transfer/frozen embryo transfer

**CQ024**: Is luteal support in fresh embryo transfer effective in improving ART performance?

Answers

1. In fresh embryo transfer, luteal support using progesterone is effective for ART. (B)
2. For luteal support, the administration route of progesterone can be oral, vaginal, or intramuscular injection. (B)
3. Luteal support continues from the day of egg collection until at least the day of the pregnancy test. (B)

**CQ025**: How safe is fresh embryo transfer?

Answers

1. Fresh embryo transfer has cumulative pregnancy and live birth rates comparable to those for frozen/thawed embryo transfer. (B)
2. If an increase in blood progesterone level is observed at the time of egg collection, avoid fresh embryo transfer and perform frozen embryo transfer. (B)
3. If a thin endometrium is observed at the time of egg collection, avoid fresh embryo transfer and perform frozen embryo transfer. (C)

**CQ026**: What is the effectiveness and safety of frozen/thawed embryo transfer? Is frozen embryo transfer more effective than fresh embryo transfer?

Answers

1. In high responders, the first frozen embryo transfer following the freeze‐all strategy may increase live birth rate compared to fresh embryo transfer. (B)
2. It has been reported that frozen embryo transfer may affect fetal development and the incidence of maternal pregnancy complications. (B)
3. The freeze‐all protocol is performed for cases where the implementation of this method is considered to be beneficial. (A)

**CQ027**: Is the hormone therapy cycle in frozen/thawed embryo transfer superior to the natural cycle?

Answers

1. The hormone therapy cycle has the same effectiveness as the natural cycle in terms of pregnancy rate and live birth rate. (A)
2. In the hormone therapy cycle, an effectiveness equivalent to that in the natural cycle can be obtained by appropriately setting the administration route, administration method, and dosages of the estrogen and progesterone. (B)

**CQ028**: What are the requirements and precautions for facilities that cryopreserve gametes, embryos, and ovaries?

Answer

1. At facilities that freeze and store human gametes, embryos, ovarian tissues, etc., the following guidelines apply:

- It must be an ART implementation registration facility and have been reviewed and approved by the Ethics Committee. (A)
- The storage container installation room must be lockable. (A)
- An equipment monitoring system must be established for the amount of liquid nitrogen in the cryopreservation container and temperature changes due to transpiration. (B)
- The facility must establish a safety management system for hypoxia in storage facilities due to liquid nitrogen evaporation. (B)
- A system must be built to reliably retain cryopreservation records for the required period. (A)
- The facility must screen for infectious diseases, such as human immunodeficiency virus, hepatitis B virus, and hepatitis C virus, at the time of freezing. (B)
- The facility must thoroughly consider and implement measures against power outages. (B)

### 3.7 Chapter G. Add‐on medical treatment

**CQ029**: Is the endometrial embryo receptivity test effective for repeated implantation failure?

Answers

1. Individualized embryo transfer is based on the results of an endometrial embryo receptivity test in patients with repeated implantation failure. (C)
2. In cases other than those with repeated implantation failure, individualized embryo transfer is performed in cases where the number of embryos that can be obtained is limited. (C)

**CQ030**: Is the endometrial microbiome test effective in improving ART performance?

Answers

1. The effectiveness of the endometrial microbiome test in infertility treatment is yet to be clarified. (C)
2. There is no established treatment to predominate the genus *Lactobacillus* in the presence of changes in the endometrial microbiota. (C)
3. There are several methods for collecting endometrial fluid for the examination of the endometrial microbiota, none of which are problematic from the viewpoint of patient safety. (C)

**CQ031**: Is the stimulation of endometrium embryo transfer (SEET) method effective for repeated implantation failure?

Answers

1. It is unclear at this time whether the SEET method improves clinical pregnancy rates in repeated implantation failure compared with those in the untreated group. However, there are some reports that the SEET method improved the clinical pregnancy rate, so it is considered as a treatment option. (C)
2. Compared to those in the untreated groups, no differences in the occurrences of adverse events, such as miscarriage, premature birth, multiple pregnancies, ectopic pregnancy, and fetal malformations (chromosomal abnormalities, morphological abnormalities, and anatomical abnormalities) were observed in the SEET‐treated group with repeated implantation failure. (B)
3. It is unclear whether the SEET method improves clinical pregnancy rates, not only in cases of repeated implantation failure. However, there are reports suggesting the effectiveness of the SEET method, so it is considered as a treatment option. (C)

**CQ032**: Is type 1 T helper (Th1)/type 2 T helper (Th1) measurement recommended for repeated implantation failure?

Answers

1. Th1/Th2 measurements using peripheral blood may be useful in diagnosing repeated implantation failure. (C)
2. Th1/Th2 measurements using the endometrium may be useful in diagnosing repeated implantation failure. (C)
3. If embryos of good quality fail to implant following several embryo transfers, consider a peripheral blood Th1/Th2 ratio test. (C)

**CQ033**: Is a transfer medium containing high concentration of hyaluronic acid effective for repeated implantation failure?

Answers

1. It has been shown that the addition of high concentration hyaluronic acid as an adhesive compound to the embryo transfer medium improves clinical pregnancy and live birth rates. (B)
2. The use of embryo transfer media containing high concentrations of hyaluronic acid in patients with repeated implantation failure may improve ART outcomes. (B)

**CQ034**: Is endometrial scratching effective for improving ART outcome?

Answers

1. The effect of endometrial scratches on implantation has not been determined. (C)
2. There are no fixed views on the target patients, the method of approaching the endometrium, the timing of treatment, and the number of treatments. (C)

**CQ035**: Is immunotherapy such as tacrolimus/low dose aspirin effective for repeated implantation failure?

Answers

1. Treatment with low dose aspirin/glucocorticoids for repeated implantation failure may be effective. (C)
2. The use of heparin, tacrolimus, hydroxychloroquine, immunoglobulins, fat emulsions, TNF inhibitors, etc., is considered as a treatment for repeated implantation failure. (C)

### 3.8 Chapter H. Psychological support and counseling

**CQ036**: What information and mental support do patients undergoing fertility treatment need? Are psychological and educational interventions effective in psychosocial assessment and support of infertile patients?

Answers

1. Provide infertility patients (couples) with general and institution‐specific information on infertility treatment. (B)
2. Detect the needs and wishes of infertile patients and share these with the medical staff to respond. (B)
3. Consider providing psychological support to infertile patients who need or are deemed to need it. (B)
4. Psychological and educational interventions improve the mental health of infertile patients who need such interventions. (B)
5. It is unclear whether psychological and educational interventions improve pregnancy outcomes in infertile patients. (C)

### 3.9 Chapter I. Male infertility

**CQ037**: Is the Y‐chromosome microdeletion test recommended before performing testicular sperm extraction (TESE)?

Answers

1. Perform a Y‐chromosome microdeletion test before microdissection testicular sperm extraction (micro‐TESE). (A)
2. If a microdeletion of the Y chromosome is found, create an environment where patients can receive genetic counseling if they so desire. (B)

**CQ038**: Are phosphodiesterase 5 (PDE5) inhibitors effective against male infertility with erectile dysfunction?

Answer

1. PDE5 inhibitors are effective against male infertility with erectile dysfunction. (A)

**CQ039**: Is clomiphene citrate (CC) effective against male infertility?

Answer

1. Improvements in sperm concentration and motility can be expected with CC in oligospermia cases with low gonadotropin and testosterone levels. (B)

**CQ040**: Is amoxapine, a tricyclic antidepressant, effective in treating retrograde ejaculation?

Answer

1. Amoxapine is effective in treating retrograde ejaculation. (B)

## CONFLICT OF INTEREST

None declared.

